# Extended lymphadenectomy for high-risk prostate cancer in patients with chronic lymphocytic leukemia may not be necessary: a report of two cases

**DOI:** 10.1186/s12885-019-5876-x

**Published:** 2019-07-09

**Authors:** Yinjie Zhu, Yanqing Wang, Zhiyu Qian, Jiahua Pan, Qiang Liu, Baijun Dong, Wei Xue

**Affiliations:** 1 Department of Urology, Renji Hospital, School of Medicine, Shanghai Jiao Tong University, 1630 Dongfang Road, Pudong District, Shanghai, 200127 People’s Republic of China; 2 David Geffen School of Medicine at University of California Los Angeles, Los Angeles, CA USA; 3 Department of Pathology, Renji Hospital, School of Medicine, Shanghai Jiao Tong University, Shanghai, China

**Keywords:** Prostate neoplasms, Chronic lymphocytic leukemia, Lymphadenectomy, Positron emission tomography/computed tomography

## Abstract

**Background:**

Chronic lymphocytic leukemia is a malignancy with good prognosis. However, the incidence of secondary tumors increases every year after the diagnosis of chronic lymphotcytic leukemia. One of the induced secondary tumors is prostate cancer. For high-risk prostate cancer in particular, the standard therapy is radical prostatectomy and extended lymphadenectomy, which carries high risks of lymphatic leakage and reduced quality of life. Currently, there has been no study reporting the necessity of extended lymphadenectomy for high-risk prostate cancer in patients with chronic lymphocytic leukemia.

**Case presentation:**

We reported two cases with concomitant high-risk prostate cancer and chronic lymphocytic leukemia. The first patient was a 60-year-old male diagnosed with synchronous prostate cancer and chronic lymphocytic leukemia. The second patient was a 70-year-old male initially presented with chronic lymphocytic leukemia alone but was then diagnosed with high-risk prostate cancer nine years later. Both patients received neoadjuvant androgen deprivation therapy and robot-assisted radical prostatectomy. The first patient underwent extended lymphadenectomy and developed prolonged postoperative lymphatic cyst. Histology showed chronic lymphocytic leukemia infiltration in resected lymph nodes. Serum prostate-specific antigen levels at one and 13 months post-operation were both 0.01 ng/ml. The second patient received positron emission tomography/computed tomography before androgen deprivation therapy, which showed mild fluorodeoxyglucose-avidity in lymph nodes across the entire body. Lymph node biopsy showed only chronic lymphocytic leukemia. The patient experienced no postoperative complication. Serum prostate-specific antigen levels at one and nine months post-operation were both 0.02 ng/ml.

**Conclusions:**

Extended lymphadenectomy may not be necessary for patients with concomitant high-risk prostate cancer and chronic lymphocytic leukemia, but such patients must undergo thorough preoperative assessment and mindful postoperative follow-up. Positron emission tomography/computed tomography may be valuable in detecting nodal metastases. A lymph node biopsy is necessary for patients with an ambiguous positron emission tomography/computed tomography in the metastatic involvement of lymph node.

## Background

Chronic lymphocytic leukemia (CLL) is a mature B lymphocyte-derived indolent lymphoma, resulting in diffuse lymphadenopathy. The treatments for CLL include chemotherapy, targeted therapy, and symptomatic treatment [1], while surgery is generally not recommended. In the past decade, overall survival of patients with CLL was improved thanks to the advance of targeted therapy and novel treatments. However, the incidence of induced secondary tumors is increasing annually due to the improved overall survival of CLL. Tsimberidou AM et al. [2] reviewed the data from patients with CLL at the University of Texas M.D. Anderson Cancer Center from 1985 to 2005 and found that 16% of all patients had a history of other cancers and 11.2% developed other malignancies. The most common secondary tumor was lung cancer (30%), followed by prostate cancer (13%). Falchi L et al. [3] reported that the overall prevalence of secondary cancers was 36% in patients with CLL, among which the most common secondary cancer is non-melanoma skin cancer followed by prostate cancer.

Prostate cancer is the most common malignancy of male genitourinary system, with the highest incidence in western countries. Radical prostatectomy with extended lymphadenectomy is the standard therapy for high-risk prostate cancer [4], yet extended lymphadenectomy can lead to complications of lymphatic leakage, as well as reduced quality of life. The necessity of extended lymphadenectomy for high-risk prostate cancer in patients with CLL is debatable, as lymphedema tends to be too systemic for complete resection. Moreover, long-term expansion of lymphatic vessels from CLL may cause higher incidence of lymphatic leakage, longer recovery time, and significantly reduced quality of life after extended lymphadenectomy.

Several researchers have reported some cases with concomitant prostate cancer and CLL. However, the majority of CLLs were incidentally found at the time of prostatectomy and lymphadenectomy [5–8]. No study has been done to report the necessity of extended lymphadenectomy for patients with concomitant high-risk prostate cancer and CLL. In this study, we reported two cases of patients with concomitant high-risk prostate cancer and CLL and evaluated the necessity of extended lymphadenectomy for such patients.

### Case presentation

#### Case 1

**Fig. 1:**
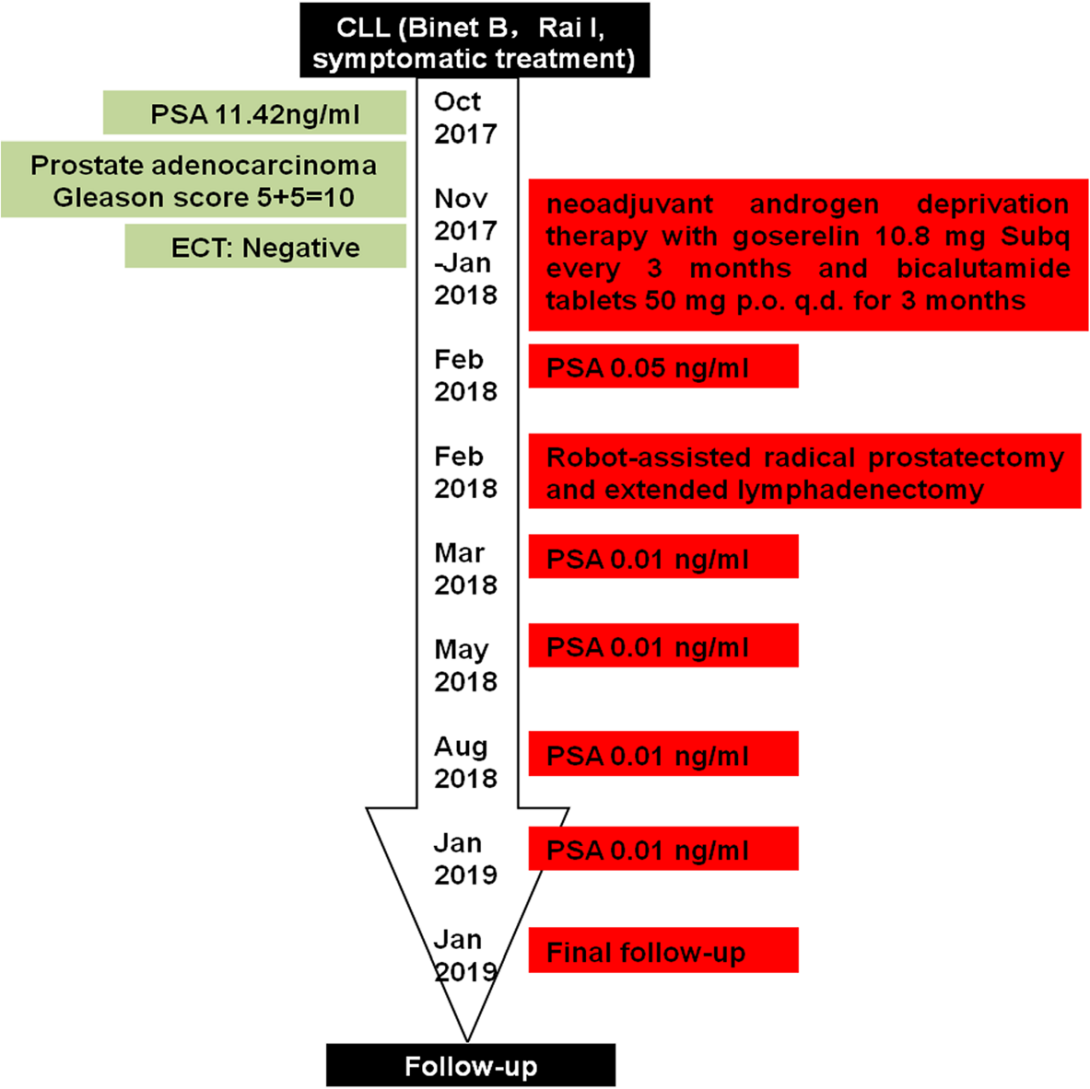
Timeline for case 1

The first patient was a 60-year-old male who was diagnosed with CLL (Binet B, Rai I) in October 2017 and recovered after symptomatic treatment. His serum prostate-specific antigen (PSA) level was elevated while being treated for CLL (11.42 ng/ml). Pelvic enhanced magnetic resonance imaging scan showed abnormal signals in the left posterior peripheral zone of prostate and diffuse lymphedema in pelvic cavity. Prostate biopsy showed a Gleason score of 5 + 5 = 10, indicative of high-risk prostate cancer with a 36% probability of lymph-node involvement according to the “Partin Tables”. Bone emission computed tomography scan was normal. Patient received neoadjuvant androgen deprivation therapy with goserelin 10.8 mg Subq every three months and bicalutamide tablets 50 mg p.o. q.d. for three months prior to his robot-assisted radical prostatectomy and extended lymphadenectomy in February 2018. His preoperative serum PSA level was 0.05 ng/ml. The prostatic envelope of the patient was intact. Diffuse lymphedema was observed in the patient’s pelvic cavity during surgery. Prostate pathology showed fibrosis, foam cell response, and a small amount of cancer tissue. The immunohistochemistry of lymph nodes showed CK(−), CD20(+), CD79a(+), CD3(−), CD5(−/+), CD21(+), CD23(+), Bcl2(+), Bcl6(−), CD10(−), Cyclind1(−), and Ki67(20%), which leads to the consideration of CLL. Patient developed prolonged lymphatic cyst and recovered after drainage. He was discharged one month later. His catheter was removed seven days after surgery. However, the patient still uses four pieces of urine pad per day to date. Follow-up serum PSA levels were all 0.01 ng/ml for one, three, six, nine and 13 months after surgery (Fig. 1).

#### Case 2

**Fig. 2:**
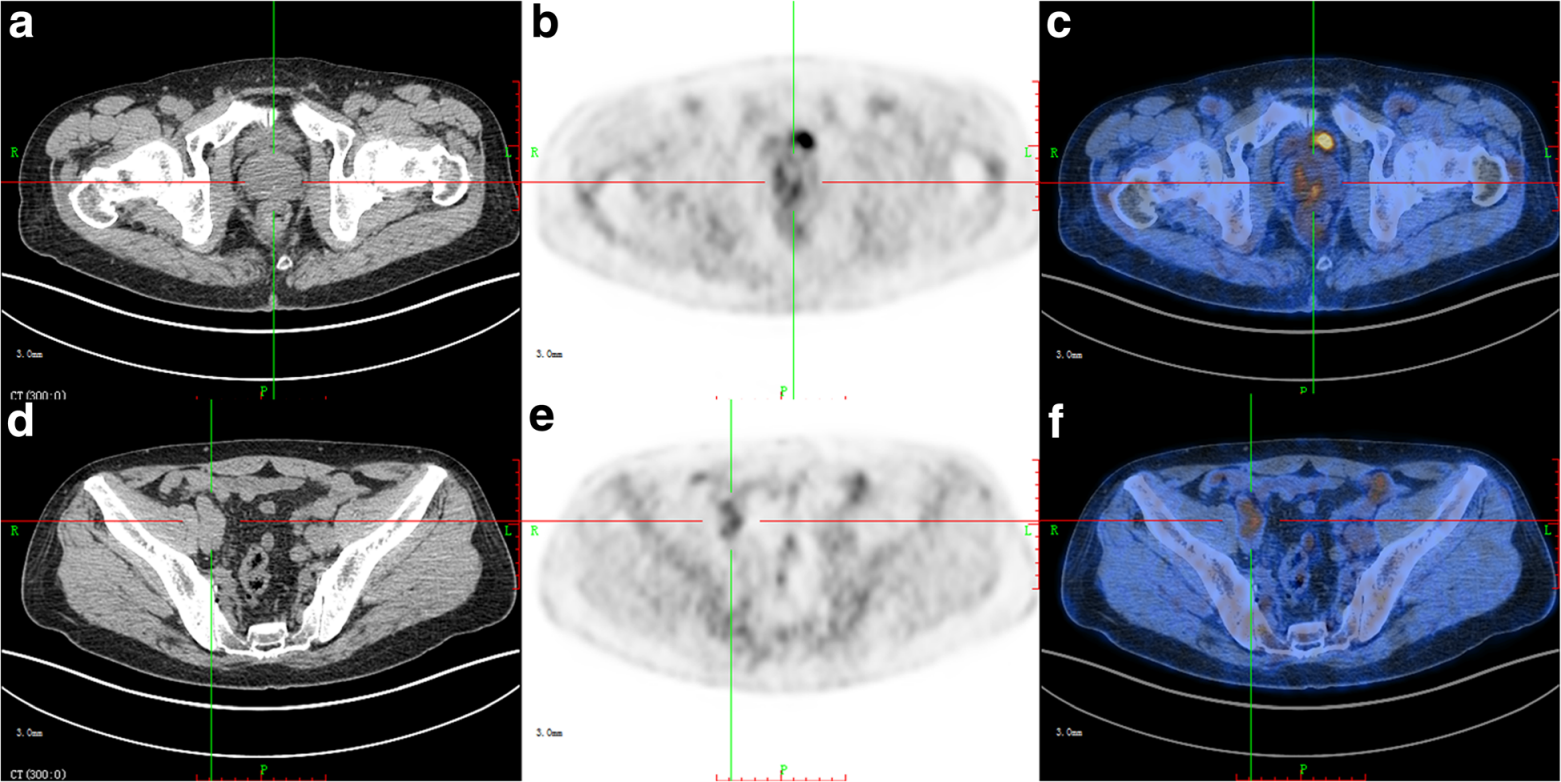
Positron emission tomography/computed tomography (PET/CT) scan of pelvic cavity of the second patient. **a**. Non-contrast CT scan of the prostate, **b**. PET scan of the prostate, **c**. Fused PET/CT scan of the prostate, **d**. Non-contrast CT scan of the lymph nodes in the pelvic cavity, **e**. PET scan of the lymph nodes in the pelvic cavity, **f**. Fused PET/CT scan of the lymph nodes in the pelvic cavity

**Fig. 3:**
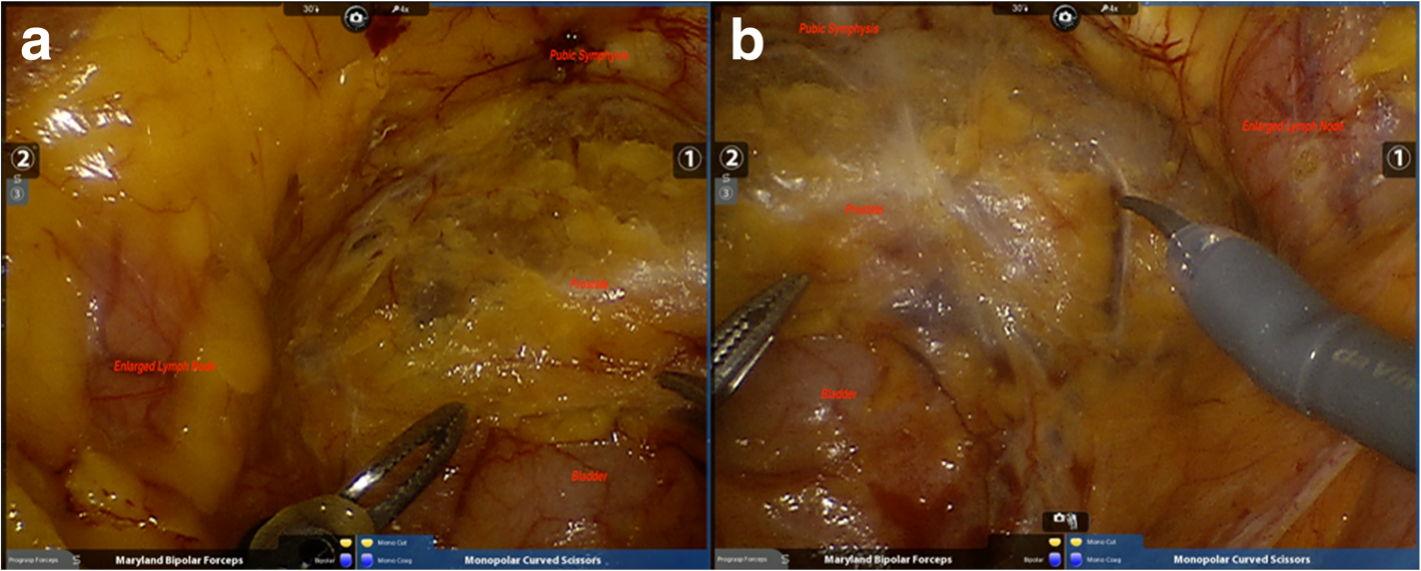
Intraoperative findings of pelvic cavity of the second patient under through robot. **a**. Left pelvic cavity, **b**. Right pelvic cavity

**Fig. 4:**
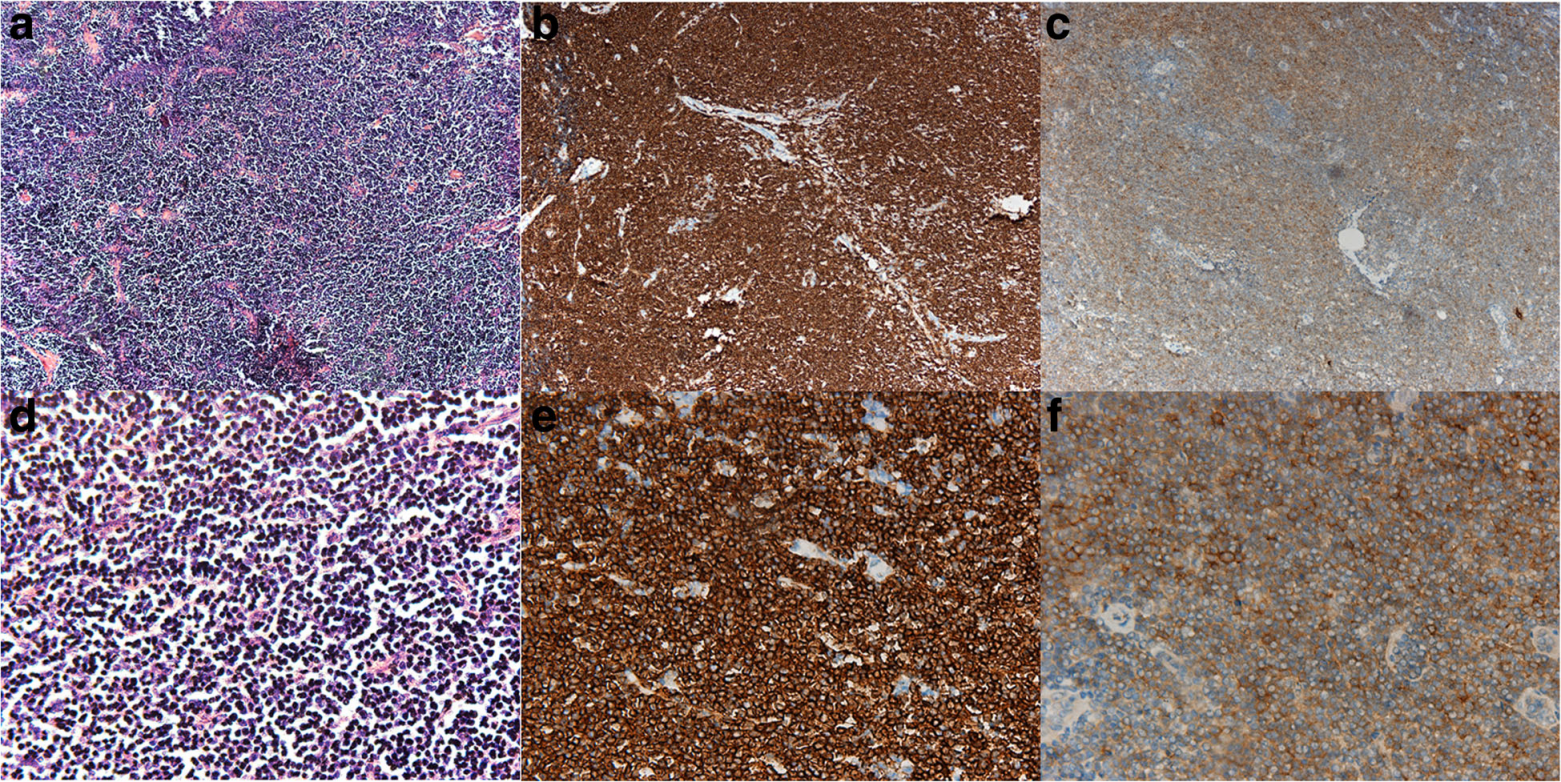
Pathology of the lymph nodes in the pelvic cavity. **a**. Hematoxylin-eosin stain with original magnification 10×, **b**. CD20 stain with original magnification 10×, **c**. CD23 stain with original magnification 10×, **d**. Hematoxylin-eosin stain with original magnification 40×, **e**. CD20 stain with original magnification 40×, **f**. CD23 stain with original magnification 40×

**Fig. 5:**
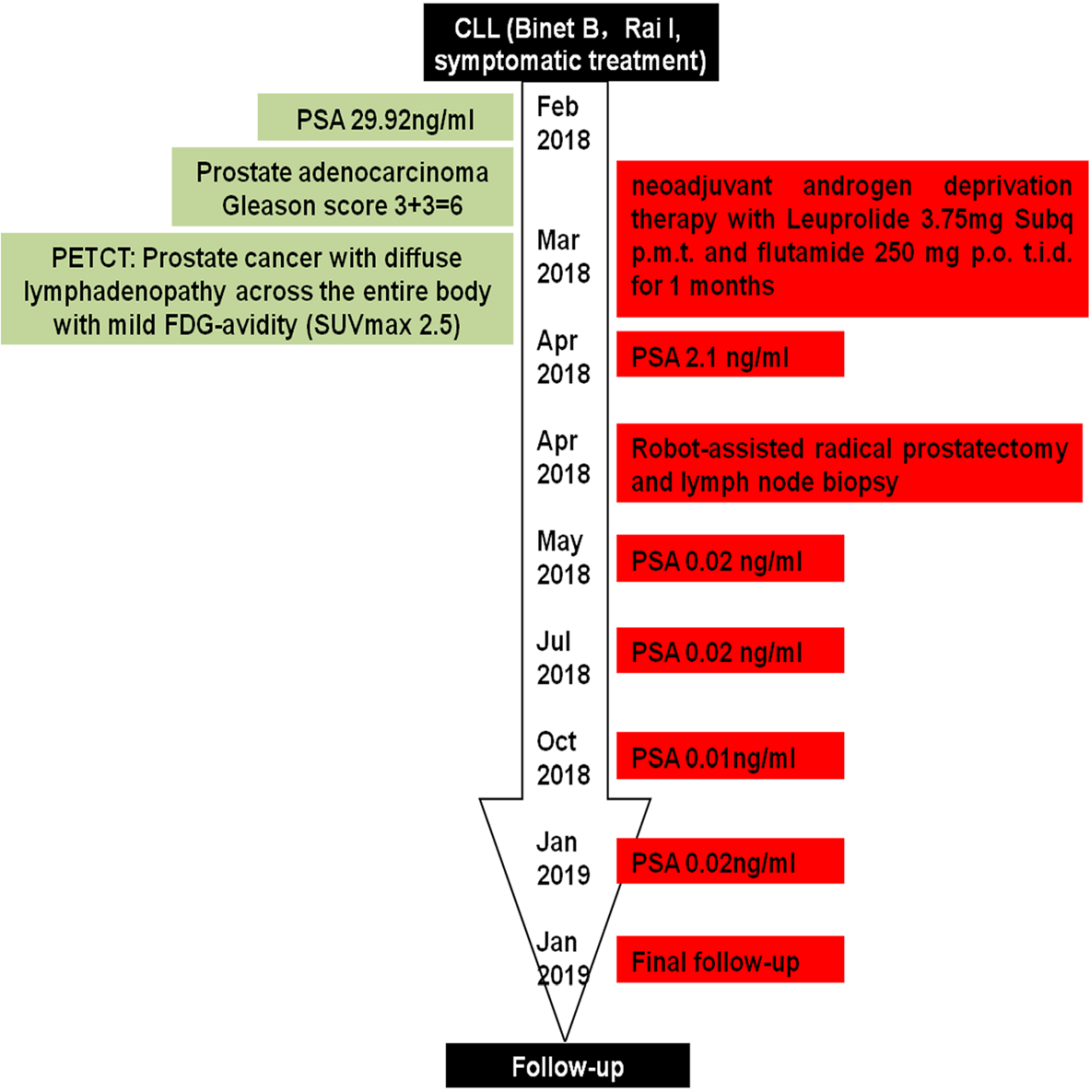
Timeline for case 2

The second patient was a 70-year-old male who was diagnosed with CLL (Binet B, Rai I) in May 2009. He recovered after symptomatic treatment and did not recrudesce or receive additional therapy. His serum PSA level was found elevated during routine screening (29.92 ng/ml). Pelvic enhanced magnetic resonance imaging scan showed abnormal signals in the left peripheral zone, middle transitional zone, and right peripheral zone of prostate gland. Magnetic resonance imaging scan also showed lymphadenopathy adjacent to bilateral iliac vessels of his pelvic cavity. A prostate biopsy established the diagnosis of prostate cancer with a Gleason score of 3 + 3 = 6 (high-risk prostate cancer). The “Partin Tables” indicated a 2% probability of lymph-node involvement. ^18^F-fluorodeoxyglucose (FDG)-positron emission tomography/computed tomography (PET/CT) showed moderate FDG-avidity in the right peripheral zone and transitional zone of prostate and diffuse lymphadenopathy across the entire body with mild FDG-avidity, of which the maximum standardized uptake value (SUVmax) was 2.5 (Fig. 2). Patient received androgen deprivation therapy (Leuprolide 3.75 mg Subq p.m.t. and flutamide 250 mg p.o. t.i.d.) for one month. Preoperative serum PSA level was 2.1 ng/ml. Robot-assisted radical prostatectomy and lymph node biopsy were performed in April 2018. The prostatic envelope of the patient was intact. Diffuse lymphedema was observed in pelvic cavity during surgery (Fig. 3). Prostate pathology showed a tumor with a maximum diameter of 1.5 cm, as well as a 3 + 4 = 7 Gleason score. The immunohistochemistry of lymph nodes showed CK(−), CD20(+), CD79a(+), CD3(−), CD5(−/+), CD21(−/+), CD23(+), Bcl2(+), Bcl6(−), CD10(−), Cyclind1(−), and Ki67(10%), considering CLL (Fig. 4). Patient experienced no perioperative complications and was discharged four days after surgery. His catheter was removed seven days post-operation. Urinary functions recovered three months post-operation. Follow-up serum PSA levels were all ≤0.02 ng/ml for one, three, six and nine months after surgery (Fig. 5).

## Discussion

To the best of our knowledge, this is the first study to report the necessity of extended lymphadenectomy for patients with concomitant high-risk prostate cancer and CLL. Our study showed that extended lymphadenectomy might not be necessary. In the two cases we reported, the first patient had synchronous prostate cancer and CLL, while the second patient was first diagnosed with CLL and then high-risk prostate cancer nine years later. CLLs were stable after symptomatic treatment in both patients. In the first case, we performed extended lymphadenectomy that resulted in prolonged lymphatic cyst; the second patient received lymph node biopsy without complications. The pathology of resected lymph nodes indicated CLL in both patients and their serum PSA levels were both ≤0.02 ng/ml nine months after surgery. Although the follow-up duration might not be long enough, together these findings indicated that both patients were cured of prostate cancer.

It was reported that the risk of the incidence of secondary tumors for CLL patients was 2.2 times higher [2], the presence of second tumors was associated with reduced survival [3], and the prevalence of second tumors was similar between patients who required CLL treatment and patients who remained untreated [3]. Additionally, the treatment regimen did not affect the risk of developing subsequent cancers [2], which indicates treating CLL does not contribute to the development of second cancers. Past studies were done to understand the cause of secondary tumors. Firstly, genetic susceptibility can be a contributing factor [9]. Additionally, shared risk factor may be an explanation for double primary tumors [2, 3], such as older age, male gender, elevated 2-microglobulin, elevated lactate dehydrogenase, elevated creatinine, and low platelet counts. CLL-induced immunological impairment was a debatable factor [2, 3, 10, 11]. Hisada et al. [10] observed a great number of Kaposi sarcomas among CLL patients but no such finding was reported by other studies [2, 3, 11].

For patients with CLL and a secondary tumor, the secondary tumor is the major cause of mortality [3]. Treatment for these patients should be comprehensive and individualized. If CLL is acute and the patient is unstable, CLL should be treated with the highest priority. Otherwise, the secondary tumor should be the main focus of the treatment. If surgery is suitable for the secondary tumor, surgery-based comprehensive therapy is recommended [12]. Radical prostatectomy can improve the prognosis of patients with high-risk prostate cancer. Although neoadjuvant androgen deprivation therapy is not recommended in the current practice for its inefficacy towards the prognosis of patients with prostate cancer, neoadjuvant androgen deprivation therapy could reduce the difficulty of surgery [4]. Therefore, we performed neoadjuvant androgen deprivation therapy followed by radical prostatectomy in both cases. However, although extended lymphadenectomy benefits patients with high-risk prostate cancer, its excessive risk of lymphatic leakage, which reduces the patient’s quality of life, is very concerning. Moreover, there are studies that do not support performing extended lymphadenectomy on patients with high-risk prostate cancer and CLL. Firstly, according to previous case reports [5–8], metastatic prostate cancer cells were not found in lymph nodes infiltrated by CLL. Additionally, extended lymphadenectomy may precipitate the progression of CLL, as it might impair the nutrition and immunity of patients. Eisenberger CF et al. [8] found eight patients with CLL among 4319 men who underwent radical prostatectomy. After a 24-month follow-up, two patients experienced disease progression and one of them required subsequent treatments. Last but not least, CLL may inhibit the progression of prostate cancer [5, 8] partially due to an increase of CD44 expression in circulation. CD44 is a transmembrane adhesion molecule whose expression is increased in lymphoma cases [5, 8], whereas its decreased expression is an indicator of prostate cancer progression/metastasis [13]. In this study, the first patient underwent extended lymphadenectomy that resulted in prolonged lymphatic cyst, while the other received lymph node biopsy without perioperative complication, and both patients were cured. Although the number of our cases was too small, and the value was not high, the literature above and the results of our cases did give us an indication that extended lymphadenectomy for patients with high-risk prostate cancer and CLL might not be necessary.

No systematic study was done to investigate the rationality of not performing lymphadenectomy on patients with CLL and a secondary tumor. Ozturk K et al. [14] reported a case with concomitant cutaneous squamous cell carcinoma and CLL and found two lymph nodes were positive for squamous cell carcinoma metastases among 25 lymph nodes involved by CLL. Tomaszewski JM et al. [15] showed 31 out of 273 lymph nodes involved in CLL were infiltrated by squamous cell carcinoma cells in five cases of concomitant cutaneous squamous cell carcinoma and CLL. These two studies hinted that it was unreasonable to not perform lymphadenectomy for patients with CLL and a secondary tumor. Thus, it is important to investigate how to determine whether to undergo lymphadenectomy and the range of lymphadenectomy. It was reported that PET/CT was valuable for nodal staging [14, 15]. The SUVmax was > 10 for the lymph nodes with tumor metastases, whereas the SUVmax was ≤2.5 for the lymph nodes with CLL. For the second case in our study, the SUVmax was 2.5 for the swollen lymph nodes and its pathology was CLL, which was consistent with results of literatures. Additionally, although the second patient only received a lymph node biopsy, he was cured. This indicated the undissected lymph nodes weren’t infiltrated by prostate cancer cells and suggested PET/CT to be a valuable approach in detecting nodal metastasis. With the advance of molecular biology research, new imaging agents such as ^11^C-choline and ^68^Ga-prostate-specific membrane antigen were reported to be more accurate in the diagnosis and recurrence evaluation of prostate cancer than ^18^F-FDG [16]. These new imaging methods may have a higher value in the evaluation of enlarged lymph nodes in patients with prostate cancer and CLL, but further research is needed.

## Conclusions

Extended lymphadenectomy may not be necessary for patients with concomitant high-risk prostate cancer and CLL, but such patients must perform complete preoperative assessment and rigorous postoperative follow-up. PET/CT may be valuable in the differentiation of nodal metastases, a lymph nodes biopsy is necessary for patients with an ambiguous PET/CT in the metastatic involvement of lymph node.

## Data Availability

The data is publicly available or can be requested.

## Abbreviations

CLL: Chronic lymphocytic leukemia
FDG: Fluorodeoxyglucose
PET/CT: Positron emission tomography/computed tomography
PSA: Prostate-specific antigen
SUV: Standardized uptake value
